# Selective and Efficient Synthesis of Pine Sterol Esters Catalyzed by Deep Eutectic Solvent

**DOI:** 10.3390/molecules28030993

**Published:** 2023-01-19

**Authors:** Honggang Shi, Zeping Lu, Huajin Xu, Shushu Wang, Binbin Nian, Yi Hu

**Affiliations:** State Key Laboratory of Materials-Oriented Chemical Engineering, School of Pharmaceutical Sciences, Nanjing Tech University, Nanjing 210009, China

**Keywords:** deep eutectic solvents, pine sterol esters, esterification, selectivity synthesis

## Abstract

Phytosterol esters have attracted widespread academic and industrial interests due to their advantages in lowering cholesterol, as antioxidants, and in preventing or treating cancer. However, the generation of by-products limits the application of phytosterol esters in food fields. In this study, deep eutectic solvents (DESs), a series of green, nontoxic, low-cost and biodegradable solvents, were adopted as the catalyst for the synthesis of pine sterol esters. The results showed that the acidic DES which was prepared with choline chloride (ChCl) and p-toluene sulfonic acid monohydrate (PTSA) with a molar ratio of 1:3 performed best in the prescreening experiments. To further improve the efficiency of the pine sterol ester, the molar ratio of substrates, the amount of catalyst, the reaction temperature and the reaction time were optimized, and its yield was improved to 94.1%. Moreover, the by-products of the dehydration side reactions of the sterol can be efficiently inhibited. To make this strategy more universal, other fatty acids were also used as the substrate for the synthesis of pine sterol esters, and a yield of above 92.0% was obtained. In addition, the reusability of DES was also investigated in this study, and the efficiency of DES was well maintained within five recycled uses. Finally, DFT calculations suggested that the suitable H-bonds between ChCl and PTSA decreased the nucleophilic capacity and increased the steric hindrance of the latter, and further prevented the attack on ^β^H and reduced the generation of by-products. This study developed a reliable and eco-friendly strategy for the preparation of high-quality phytosterol esters with low-dosage catalyst usage and high selectivity.

## 1. Introduction

As one of the 12 principles of green chemistry, the utilization of renewable feedstock as a succedaneum of the petrochemical base has always been always a key strategy to make a sustainable and green chemistry process in contemporary industries [1,2,3]. Moreover, the utilization of natural starting materials as an alternative to primary resources is of growing interesting and has attracted more attention in health-related fields [4]. In view of the great advantages of phytosterols in preventing hypertension, hyperlipidemia, and other cardiovascular and cerebrovascular diseases, they have become one of the most consumed food additives [5,6]. Phytosterols are a series of sterols present in plants which have been shown to block cholesterol absorption sites in the human gut, thus helping to reduce cholesterol levels in humans. For instance, Moreau and coworkers suggested that phytosterols play a key role in stabilizing the structure of plant cell membranes and protecting the cell from oxidation [7]. Moreover, due to their chemical structure being similar to that of cholesterol, phytosterols can also competitively inhibit the absorption of cholesterol, and thereby lower serum cholesterol levels [8]. For example, Katan et al. indicated that a daily intake of 2 to 3 g of phytosterols can reduce low-density lipoprotein cholesterol (LDL-c) levels in the blood by approximately 10% [9]. Most recently, phytosterols have been found to have antioxidant, anti-inflammatory, antitumor and other physiological activities [8], and have been used as cholesterol-lowering agents marketed as drugs, dietary supplements and food additives. Although phytosterols have many fascinating advantages, their practical application in the food industry has been limited by their drawbacks, such as a high melting point and poor oil solubility [10,11]. To address these problems, phytosterol esters, which demonstrate almost similar physiological function as phytosterols, are proposed as an alternative to phytosterols due to their significantly lower melting point and higher oil solubility [11]. Phytosterol esters have solubility properties comparable to those of edible fats and oils, which facilitates their incorporation into foods with hydrophobic matrices and increases the bioavailability of phytosterols. Esterified phytosterols have been shown to have a higher cholesterol-lowering effect. In the Plat et al. study, instead of consuming 6 g of phytosterols, only 2–3 g of phytosterol esters were required to reduce serum cholesterol by 10% [12].

**Scheme 1:**
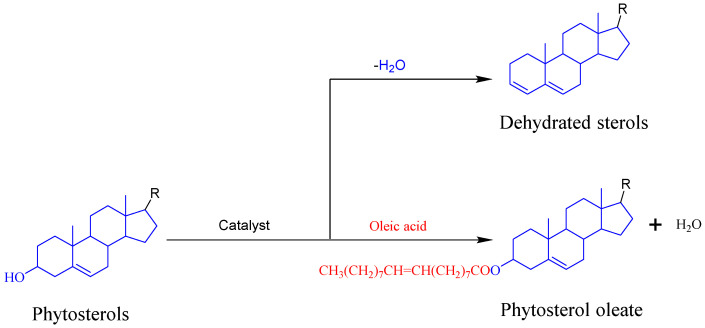
Esterification of pine sterols and its dehydration side reaction.

The pathway of the esterification of phytosterols is displayed in Scheme 1. The main competitive reaction of this process is the dehydration of sterols at high temperatures [13]. At present, the main methods to synthesize fatty acid esters of phytosterols are chemical and enzymatic. Although the enzymatic synthesis of phytosterol fatty acid esters has the advantages of mild conditions and green environmental protection, its industrial application is limited by low efficiency and high cost [14,15,16,17]. Therefore, chemical synthesis is still the most widely used method for the preparation of phytosterol esters. Many researchers are committed to developing different catalysts to improve the yield of phytosterol esters and the selectivity of esterification, such as potassium bisulfate [18], sulfuric acid [19], MgO [20] and acidic ionic liquids [13,21,22]. Although these existing chemical catalysts have advantages in terms of their reaction speed or yield, they are still plagued by corrosive equipment, low reaction selectivity, high energy consumption and environmental pollution. Most of these catalysts can only be used at a high temperature, which will produce a large amount of dehydration by-products and limits their applications in foods [13,23]. Moreover, the removal of solvents and catalysts is also a major barrier preventing their industrial applications [24].

In recent years, as a new generation of ionic liquids, deep eutectic solvents (DESs) have attracted extensive attention. DESs are composed of quaternary ammonium or phosphonium salts acting as hydrogen-bond acceptors (HBAs) combined with hydrogen-bond donors (HBDs) such as amides, polyols or carboxylic acids [25]. Different components form a stable hydrogen bond network through hydrogen bond interaction, which usually makes the melting point of the mixture lower than that of each pure component [26]. Compared with traditional ionic liquids, DESs are much cheaper, more easily synthesized, nontoxic and biodegradable. Since DESs can contain acidic components, they have also been used in small doses as catalysts in the field of biocatalysis [27,28,29,30]. In the study of Adeeb Hayyan et al., the free fatty acid content of acid crude palm oil was converted to fatty acid methyl esters for the first time using a deep eutectic solvent based on choline chloride (ChCl-DES), and the use of ChCl-DES as a catalyst in the pretreatment stage reduced the fatty acid from 9.00% to 1% [28]. Liu et al. used TOAB/PTSA as catalyst and cooked vegetable oil and waste vegetable oil as raw materials for preparing biodiesel and obtained a yield of 99.2% [29]. However, to our knowledge, no studies have been reported on the use of DESs as a catalyst for the synthesis of fatty acid esters of phytosterols.

In our previous study, the SO_3_H-functionalized ionic liquid was applied as the catalyst for the synthesis of phytosterol esters. We found that the suitable acidity of the ionic liquid can promote the esterification of phytosterols more efficiently and selectively than most of the reported chemical catalysts. However, some drawbacks such as the high consumption of catalysts, high reaction temperature and dark color of the resulting products made this strategy rarely used on an industrial scale. Inspired by this and the urgent need to develop an efficient and sustainable strategy of phytosterol esters production, in this study, DESs were applied as the catalysts with the aim of developing a more low-cost, sustainable and efficient phytosterol esters production strategy under a mild condition.

## 2. Results and Discussion

### 2.1. Characterization of Products

**Figure 1:**
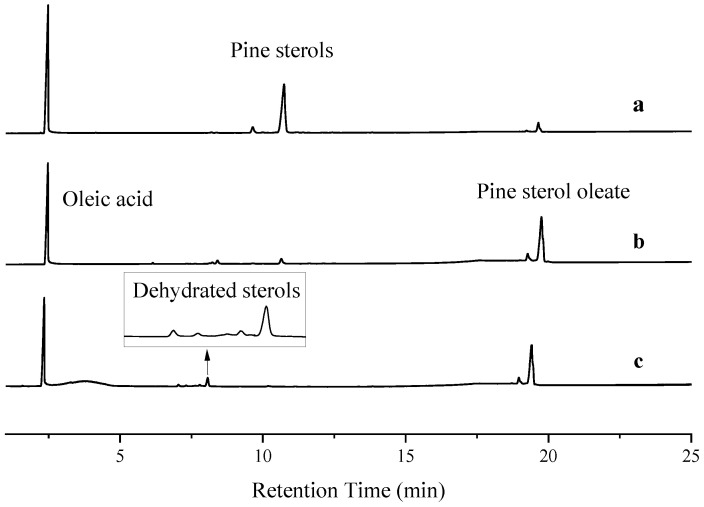
GC chromatogram of the reaction mixture using different catalysts. Reaction conditions: 2 mmol pine sterols, 4 mmol oleic acid, 7% catalyst (based on the mass of pine sterols), 120 °C, 5 h. Catalyst: (**a**) None; (**b**) DES; (**c**) PTSA.

The GC spectrum of crude products catalyzed by different catalysts was summarized as shown in Figure 1. It can be seen that pine sterol esters are successfully synthesized in both DES and free PTSA systems, while they cannot be synthesized to any appreciable extent in the absence of the catalyst. Interestingly, dehydrated sterols, which are one of the most common by-products created during the synthesis of phytosterol esters and seriously affect product quality, can only be generated in trace amounts in the DES system, while they are significantly increased in the pure PTSA system. These results indicated that DES performed better in selectivity than that of pure PTSA. Moreover, as shown in Figure S1, the results of GC-MS are consistent with our previous work [13]. This provides us with more information about the products; Figure S1a,b, represent the two major esterification products of pine sterol—campesterol and β-sitosterol—respectively. It is difficult to observe the molecular ion peaks of sterol esters in MS because of their unstable structure. Moreover, *m/z* 382 and *m/z* 396 represent the sterol peaks of campesterol oleate and β-sitosterol oleate, and the peak of *m/z* 281 can be attributed to the oleate fragment. Figure S1c,d represent the mass spectrum of the two dehydration products. Figure S1c shows the molecular ion peak of ergosta-3,5-diene at *m/z* 382, which is the dehydrated product of campesterol. Similarly, *m/z* 396 in Figure S1d is the molecular ion peak of the dehydrated product of β-sitosterol, stigmasta-3,5-diene.

### 2.2. Screening of DES Catalysts

The acidity of the hydrogen-bond donor (HBD) largely determines the acidity of DESs, which further affects the catalytic efficiency of DESs as a catalyst in esterification [31,32]. Herein, a different HBD was screened when ChCl, a common hydrogen-bond acceptor (HBA) in the related study, was used as the HBA. In this context, 16 entries of screening experiments were performed. As indicated in Table 1, the catalytic effect of DESs is in good agreement with the acidity of the corresponding HBDs. For instance, when weak organic acids were used as the HBDs, all DESs can only catalyze the reaction to no more than 50% yield. The same results are also found when other weak acids, namely metal chlorides, were used as HBDs. In contrast, as shown in entries 8–15, when the strong organic acids PTSA and sulfosalicylic were used as the HBDs of DESs, the pine sterols were completely converted. However, it is worth noting that, as shown in entry 9, although pine sterols were almost completely converted, the by-products—dehydrated sterols—account for as much as 31.1% of the products. This can be attributed to the excessive addition of an acid catalyst which is also in agreement with our previous study in cases of ionic liquids [13]. To prevent the generation of dehydrated sterols, we further reduced the amount of catalyst. As shown in entry 10, when the dosage of sulfosalicylic-based DES was reduced to 1% (*w*/*w* of pine sterols, the yield of the by-product was reduced to 3.6 ± 0.6%. Fortunately, when the dosage of PTSA-based DES was reduced to 1% (*w*/*w* of pine sterols) (*w*/*w*), the yield of by-product dehydrated sterols was reduced to 4.6 ± 0.5%, and the yield of sterol esters was increased to 90% at the same time (entry 11). The use of a high-dose catalyst will not only increase the cost, but also affect the product safety of food sterol esters. Moreover, as shown in entries 12 and 13, although TBAC/PTSA and TBAB/PTSA DESs can also catalyze the reaction to similar extent, the dark color of the products makes them unsuitable for high-quality food applications [33]. In this context, PTSA was considered as a suitable HBD of DESs for catalyzing the esterification of pine sterols and should be used for further investigation.

The molar ratio of HBA to HBD of DESs can significantly affect their physical properties and catalytic preformation. As shown in Entries 11, 14 and 15, the sterol conversion was significantly increased with the increase in the molar ratio of PTSA in DESs. However, a closer look at these reactions tells us that the high molar ratio of PTSA can also lead to a low reaction selectivity and dark color of products. Moreover, as a control, the pure PTSA was also used as a catalyst (entry 16); the results indicated that although pine sterols can also be completely converted, the by-product is as high as 8.5%, and the product was darker in color than ChCl/PTSA (1:3) (Figure S2). Similar results were obtained even when we used the same acid amount of PTSA as in ChCl/PTSA (1:3) (entry 17). This indicates that the PTSA plays a key role in the catalyzation of the esterification of pine sterols, and the formation of DES can improve its selectivity and product quality. Moreover, the pure PTSA suffers from the drawback of recycling, which can lead to environmental pollution. Taking all the above factors into consideration, the molar ratio of ChCl to PTSA in the DES catalyst was chosen to be 1:3.

### 2.3. Optimization of Reaction Conditions

To develop a more efficient, selective and sustainable strategy for the synthesis of pine sterol esters, the molar ratio of substrates, the DES dosage and the reaction temperature and reaction time were systematically investigated.

**Figure 2:**
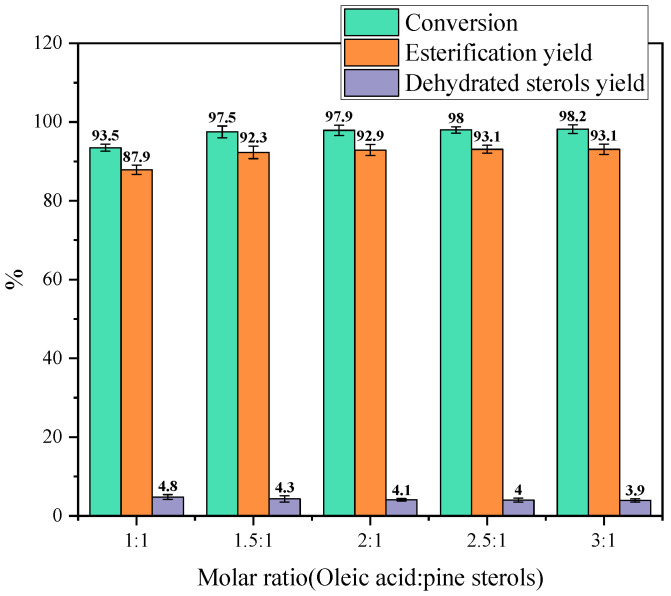
Effect of the molar ratio of oleic acid to pine sterols. Reaction conditions: 2 mmol pine sterols, 1% catalyst (based on the mass of pine sterols), 120 °C, 5 h.

As shown in Figure 2, the molar ratio of oleic acid to pine sterols (1:1–3:1) can significantly affect the yield of products. For instance, when the theoretical value (1:1) was applied, the starting material—pine sterol—cannot be completely converted (93.5%), and the yield of pine sterol esters can only reach 87.9%. Fortunately, when excessive oleic acid was added, the yield of pine sterol esters is significantly increased while the by-product, dehydrated sterol, can also be slightly decreased. This can be attributed to the fact that excessive oleic acid can be used as the reaction solvent to increase the solubility of pine sterols and reduce the viscosity of the reaction system which is conducive to the mass transfer process of the reaction system. In addition, the decrease in dehydrated sterol content is mainly due to the rapid conversion of pine sterols under the high molar ratio of oleic acid and pine sterols, which leads to the low concentration of pine sterols in the reaction mixture to hinder the occurrence of side reactions of sterol dehydration [34]. However, the yield cannot be further improved when the molar ratio of acid to alcohol increased from 1.5:1 to 3:1. Moreover, a large amount of excessive oleic acid will also increase the cost of raw materials and processes. Therefore, the 1.5:1 molar ratio of oleic acid to sterol was selected as the optimal condition.

**Figure 3:**
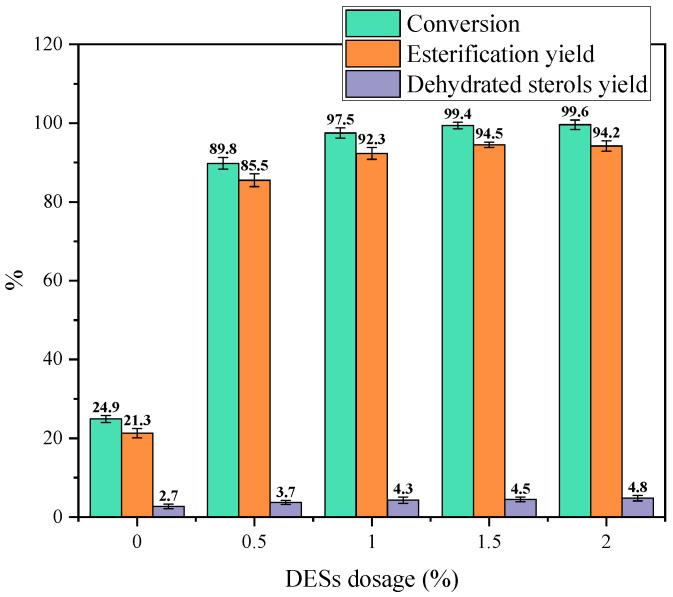
Effect of DESs dosage. Reaction conditions: 2 mmol pine sterols, 3 mmol oleic acid, 120 °C, 5 h.

In this study, the effect of DES dosage (0–2%, based on the mass of pine sterols) on the catalytic effect was investigated. As shown in Figure 3, it was observed that the dosage of DES has a significant effect on the esterification effect of pine sterol esters. Compared with the low level of reaction effects without a catalyst (product yield of 21.3%), the yield of pine sterol esters can reach 85.5% when adding 0.5% DES, indicating that the reaction effect can be significantly improved when adding a small dosage of DES catalyst. With the increase in DES dosage, pine sterols gradually tend to be completely transformed, and the yield of sterol esters reached the highest value when 1.5% DES was added. Next, the yield of sterol esters began to decrease slightly, and the yield of dehydrated sterol continued to increase when the dosage of DES was further increased, which indicated that the excessive dosage of DES catalyst was detrimental to the selectivity of the reaction (this phenomenon can be observed in the catalyst screening experiment). In addition, the lower catalyst dosage will help to reduce the production cost and product safety of sterol esters. Herein, 1.5% DES was used for the esterification of pine sterol esters.

**Figure 4:**
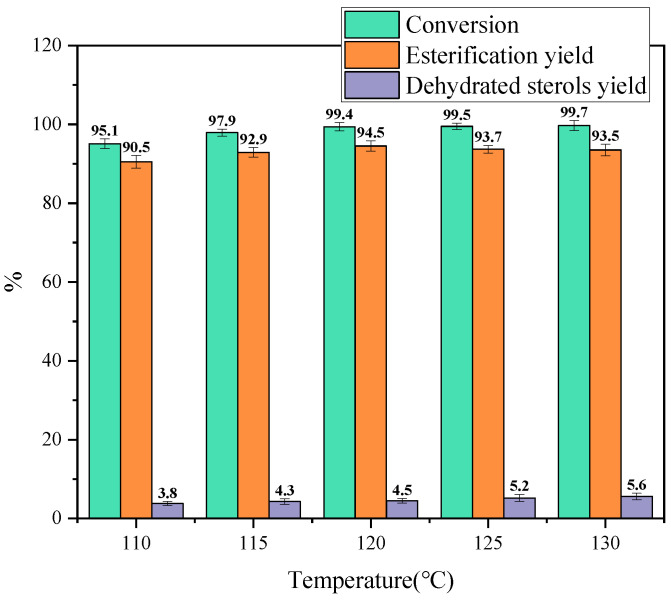
Effect of reaction temperature. Reaction conditions: 2 mmol pine sterols, 3 mmol oleic acid, 1.5% catalyst (based on the mass of pine sterols), 5 h.

The high temperature can increase the solubility of pine sterols and reduce the mass transfer resistance of the reaction system, which is favorable to the esterification reaction; however, a temperature that is too high is also prone to side reactions, so the appropriate temperature is very important for the synthesis of pine sterol esters. As shown in Figure 4, the effects of temperature on the reactions were investigated. As shown in Figure 4, the conversion and sterol esters yield were above 90% in all selected temperatures, and the highest yield (94.5%) was obtained at 120 °C. In addition, it can be observed that a high reaction temperature is also conducive to the production of dehydrated sterols. These results are in agreement with those of previous studies. Finally, 120 °C was selected for the following experiment.

**Figure 5:**
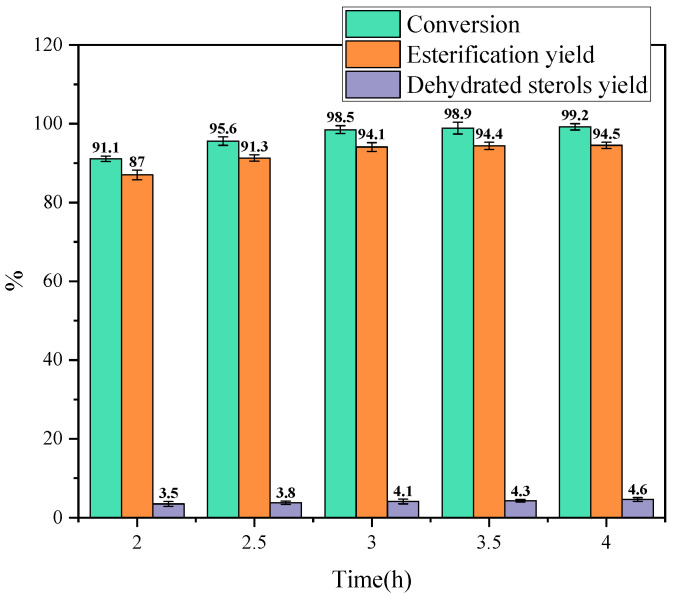
Effect of reaction time. Reaction conditions: 2 mmol pine sterols, 3 mmol oleic acid, 1.5% catalyst (based on the mass of pine sterols), 120 °C.

The effect of reaction time was summarized as shown in Figure 5. The conversion of pine sterols and product yield reached 91.1% and 87.0% at 2 h, respectively, and the growth rate gradually slowed down with the extension of time. The reaction reached completion at 3 h, and the yield of dehydrated sterols began to rise slowly with the further prolonging of the reaction time, which suggested that too long of a reaction time was detrimental to the reaction. Based on the above experimental results, 94.1% products and 4.1% dehydrated sterols were produced using 1.5% DES as the catalyst with an oleic acid and pine sterols molar ratio of 1.5:1 at 120 °C for 3 h. Compared with previous similar studies which used ionic liquid as catalysts to catalyze pine sterol esters, this study achieved the same or even better catalytic effects with a small dosage of catalysts and a more economical substrates ratio at a mild reaction temperature [13,21,22].

### 2.4. Esterification of Pine Sterols with Other Fatty Acids

In previous research, fatty acid substrates with different structures (especially the carbon chain length of fatty acids) have a great impact on the esterification results of phytostanols and phytosterols [22,35]. In this study, a variety of fatty acids with different carbon chain lengths and saturations were used for esterification with pine sterols to explore the generality of synthesis methods of sterol esters under the selected conditions. As shown in Table 2, the conversion of sterols and product yield decline with the increase in the carbon chain length of the fatty acid substrate, and there is no significant difference in the reaction results of fatty acids with different saturation; however, the yield of sterol esters corresponding to all fatty acids exceeded 93%, which indicates that the DES catalyst has a higher catalytic activity. The gas chromatographic characterization of the reaction mixture with each fatty acid as a substrate is shown in Figure S3. This shows that the synthesis method of pine sterol oleate with DES as the catalyst is suitable for the preparation of industrial phytosterol fatty acid esters with a variety of mixed fatty acids as raw materials.

### 2.5. Scale-up Validation of Reactions and Reusability of Catalysts

**Figure 6:**
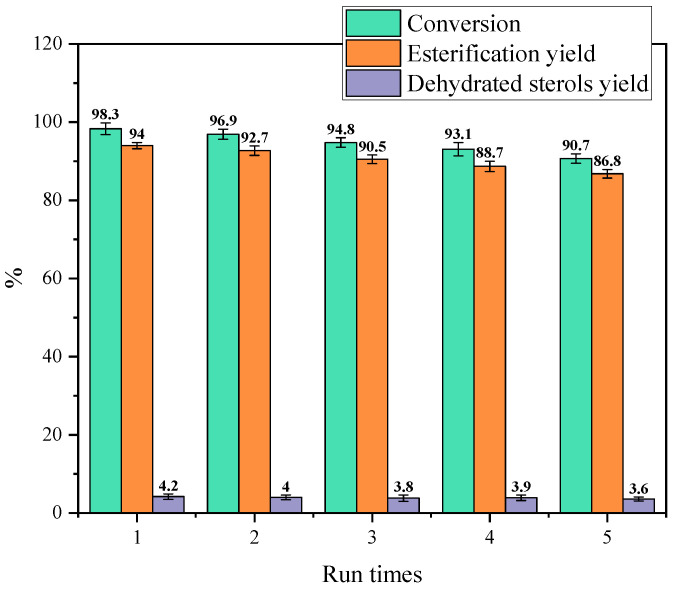
Experiments of catalyst recovery.

Considering the feasibility of the recovery operation, the DES catalyst recovery experiments were carried out in 250 mL three-neck flasks and the amount of substrate was scaled up to 10 times. The recovery of the DES catalyst in the reaction system was obtained by water washing and extraction and weighed after sufficient drying to determine the mass loss in the recovery process. As shown in Figure 6, the sterol esters yield remained at 94.0% in the reaction system that will be scaled up (first reaction cycle). In addition, the catalytic performance of the DES catalyst decreases slightly after each recycling owing to the slight loss and recovery of the catalyst during the extraction and separation process after the reaction, which can be proved by the weighing of the recovered catalyst. However, after five recoveries, the conversion and product yields still reached 90.7% and 86.8%, indicating that the DES catalyst still maintains a good performance when used at small doses.

### 2.6. Comparison with Other Chemical Methods for The Synthesis of Phytosterol Esters

Table 3 lists the reaction conditions and results of different reported chemical syntheses of phytosterol esters. As shown in the table, strong acid catalysts such as H_2_SO_4_ and NaHSO_4_ can catalyze the complete conversion of phytosterols, but they also show low esterification selectivity and will cause serious equipment corrosion and environmental pollution [19]. In addition, the catalytic effect of the solid catalyst ZnO is still very low under a high temperature (170 °C) and long reaction time (8 h) [19]. In order to avoid the influence of the catalyst on the product, the catalyst-free methods with different heating modes were also used in the synthesis of phytosterol esters in previous studies. However, in the absence of the catalyst, a large amount of excess fatty acids and high temperatures (≥180 °C) are needed to promote the reaction [11,34]. Moreover, in the catalytic experiment using a surfactant catalyst, dodecyl benzene sulphonic acid (DBSA), with different heating modes, a large number of catalysts (20 mol% of phytosterols) and complex postseparation processes are required [36,37]. Moreover, although acidic ionic liquids can also catalyze the esterification of phytosterol efficiently, the high catalyst dosage and high reaction temperature still restrict its large-scale application [13,22]. In contrast, the sterol esters synthesis method in this study adopted the green and inexpensive DES as the catalyst, which reduces the reaction cost by using less catalyst and a more economical substrate molar ratio while maintaining high catalytic activity and excellent selectivity. In addition, the low dose of the catalyst and lower reaction temperature alleviated the browning of the product and ensured its quality.

### 2.7. Reaction Mechanism of Sterols Dehydration

**Figure 7:**
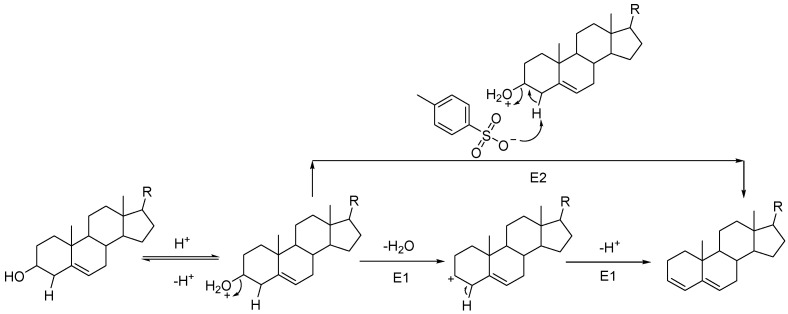
Reaction mechanism of sterol dehydration.

It is known that there are two reaction pathways for the dehydration of secondary alcohols [38]. For the dehydration of phytosterols, the reaction mechanism was shown in Figure 7. In the E1 mechanism, the hydroxyl group on the sterol is protonated by the acidic proton from the PTSA component of the DES, followed by the generation of carbenium ions through the dissociation of the C–O bond of the steroid resulting in the formation of the H_2_O leaving group. Finally, olefins are formed after the elimination of the carbocation ^β^H. However, the dehydration of sterols can proceed via a concerted step following hydroxyl protonation in the E2 mechanism, where water elimination is accomplished by simultaneous cleavage of the C–O and C–^β^H bonds with the assistance of the conjugate base anion [38,39]. It can be seen that the reaction rate of the E2 pathway of sterol dehydration is related to the ability of the conjugate base to attack ^β^H.

**Figure 8:**
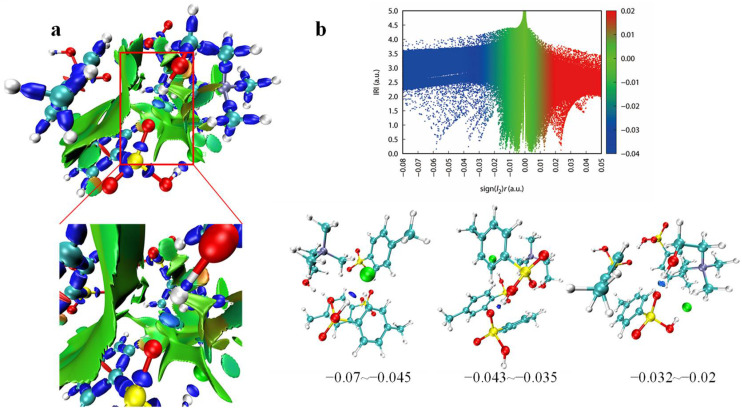
The independent gradient model based on the Hirshfeld partition (**a**), and NCI analysis of ChCl/PTSA (1:3) DES (**b**). The color from green to blue indicates the H-bonds moving from weak to strong.

Inspired by the above mechanisms, we proposed that the interactions between HBD and HBA may reduce the acidity of PTSA and provide a suitable basicity. To investigate this, the possible structure of ChCl/PTSA (1:3) DES was investigated via DFT calculations. As shown in Figure S4, eight possible geometries were listed, and the geometry in Figure S4f exhibited the lowest single point energy, which indicated that this is the most stable geometry of ChCl/PTSA (1:3). To further investigate the noncovalent interactions (NCI) and the independent gradient model based on the Hirshfeld partition (IGMH) [40], the geometry in Figure S4f was selected for further study. As shown in Figure 8, there are multiple interactions between ChCl and PTSA, with hydrogen bonding being the predominant interaction. Moreover, strong H-bonds are found between the hydroxyl group of choline and the sulfonic acid group. Figure 8a and the NCI analysis provide more information about this H-bond; its sign (λ2)ρ indicates that it is a weak H-bond. This indicates that a suitable number of electrons of the sulfonic acid group can be transferred to the hydroxyl group via this H-bond, and further decrease the basicity of the sulfonic acid group. That is to say, compared to pure PTSA, the nucleophilic capacity of ChCl/PTSA (1:3) DES was decreased, which reduces the ability of the conjugate base to attack ^β^H and prevent the reaction of the E2 pathway and further decrease the generation of by-products. Moreover, as shown in Figure 8a, PTSA was surrounded by ChCl, which indicated that the high steric hindrance would prevent the contact between PTSA and substrate subtract and thus further limit the E2 pathway of sterol dehydration. Therefore, the DES formed after the binding of ChCl by PTSA has a better esterification selectivity, which is in accordance with our previous experimental results.

## 3. Materials and Methods

### 3.1. Materials

Pine sterols (composed of 91% β-sitosterol, 7% campesterol, 0.5% stigmasterol, 0.3% brassicasterol and 1.2% others) were provided by Jiangsu KoNat Biological Products Co., Ltd. Unsaturated fatty acids (oleic acid, linoleic acid, linolenic acid) and saturated fatty acids with different chain lengths (lauric acid (C12), myristic acid (C14), palmitic acid (C16) and stearic acid (C18)) are all analytical reagent (AR) grade, which were purchased from Energy Chemical. Choline chloride (ChCl), Tetrabutylammonium chloride (TBAC), Tetrabutylammonium bromide (TBAB) and P-toluene sulfonic acid monohydrate (PTSA) were purchased from Sinopharm group, and other chemicals (including ethanol, n-hexane and petroleum ether) are AR grade and used without further purification.

### 3.2. Preparation of DESs

To prevent the possible interference of a small amount of moisture, ChCl and PTSA were vacuum dried overnight, and the two components were then mixed in a 50 mL round-bottom flask according to the specific molar ratio which is summarized in Table 4, and then stirred at 400 rpm and 60 °C for 3 h until a uniform and transparent liquid was formed. The generated ChCl/PTSA (1:3) DES exists as an off-white gel at room temperature, which can be melted into a pale-yellow liquid at 50 °C. All DESs were vacuum dried to remove moisture before being used.

### 3.3. Synthesis of Pine Sterol Esters

A 15 mL reaction tube equipped with a magnetic stirring bar was charged with pine sterol (0.8275 g, 2 mmol), different amounts of oleic acid (2–6 mmol) and DESs catalyst (0–2%, based on the mass of pine sterols). The reaction was maintained for different times (2–5 h) at different temperatures (110–130 °C) under nitrogen flow. After the reaction, a small amount of water was added to the reaction system. Then, to separate the product, the diluted reaction solution was centrifuged at 10000 rpm for 20 min, to separate the product, the pine sterol esters which cannot be dissolved in the water phase were precipitated and DESs were dissolved in the lower aqueous phase. Subsequently, the reaction mixture was used for GC analysis. Furthermore, the reaction mixture was separated by silica gel column chromatography, and the flow matching ratio was petroleum ether: ethyl acetate = 20:1 (*v*:*v*). The purified product was used for standardization. The aqueous phase mentioned above was used for recycling the DESs. The DESs dissolved in the lower water layer were recovered by the rotary evaporator and dried at 60 °C overnight for further reaction cycles.

### 3.4. Analysis of Products

The composition of the reaction mixtures was analyzed by an Agilent 6890N gas chromatograph (GC, Agilent Technologies, USA), and the parameters were the same as those in our previous work [13]. The temperature of the injection port and the detector was set to 350 °C. A DB-5ht capillary column (0.1 μm, 0.25 mm × 15 m) was equipped and the temperature was programmed from 180 °C ramp-ups to 240 °C at 10 °C/min, followed by 3 °C/min ramp-up to 260 °C, then by 20 °C/min ramp-up to 350 °C, and then by 2 °C/min ramp-up to 365 °C and finally by 10 °C/min ramp-up to 380 °C and held for 3 min. The nitrogen was used as a carrier gas at a flow rate of 1.5 mL/min and a split ratio of 20:1 (*v*/*v*). The injection volume was 1 μL. The conversion of pine sterols was calculated using the external standard method with Equation (1).

(1) $$\mathrm{Conversion}\;(\%)=\frac{\mathrm{mass}\;\mathrm{of}\;\mathrm{pine}\;\mathrm{sterols}\;\left(\mathrm{in}\right)-\mathrm{mass}\;\mathrm{of}\;\mathrm{pine}\;\mathrm{sterols}(\mathrm{out})}{\mathrm{mass}\;\mathrm{of}\;\mathrm{pine}\;\mathrm{sterols}\;\left(\mathrm{in}\right)}\times 100\%\;$$

The pine sterols were dehydrated, oxidized and undergo other side reactions at a high temperature. In order to accurately investigate the yield and reaction selectivity of pine sterol esters, this study monitored the yield of pine sterol esters and pine sterol dehydration products. The esterification yield and dehydration sterol yield were calculated by the external standard method with Equations (2) and (3), respectively:

(2) $$\mathrm{Esterification}\;\mathrm{yield}\;(\%)=\frac{\mathrm{mass}\;\mathrm{of}\;\mathrm{pine}\;\mathrm{sterol}\;\mathrm{esters}\;\mathrm{actually}\;\mathrm{generated}}{\mathrm{mass}\;\mathrm{of}\;\mathrm{all}\;\mathrm{pine}\;\mathrm{sterols}\;\mathrm{converted}\;\mathrm{to}\;\mathrm{pine}\;\mathrm{sterol}\;\mathrm{esters}}\times 100\%\;$$

(3) $$\mathrm{Dehydrated}\;\mathrm{sterols}\;\mathrm{yield}\;(\%)=\frac{\mathrm{mass}\;\mathrm{of}\;\mathrm{dehydrated}\;\mathrm{sterols}\;\mathrm{actually}\;\mathrm{generated}}{\mathrm{mass}\;\mathrm{of}\;\mathrm{all}\;\mathrm{pine}\;\mathrm{sterols}\;\mathrm{converted}\;\mathrm{to}\;\mathrm{dehydrated}\;\mathrm{sterols}}\times 100\%\;$$

The composition of the product and dehydrated sterols were determined by Agilent 7890-5977 GC-MS, which was equipped with an HP-5MS UI capillary column (30 m × 0.25 mm × 0.25 μm). The temperatures of the injection port and the ionization source were 300 °C and 200 °C, respectively. The helium (>99.996%) was used as a carrier gas with a flow rate of 1.5 mL/min and a split ratio of 20:1 (*v*/*v*). The column temperature was programmed from 200 °C increased to 320 °C and held for 60 min. The electron ionization energy was 70 eV. The injection volume was 1 μL.

### 3.5. Quantum Chemical Calculations

The geometries search processes of DES were performed according to our previous study [2]. First, the ten most possible geometries of ChCl-PTSA were searched and collected via molclus software version (1.9.9) [46]. These initial geometries were preoptimized via xTB program version 6.5.1 [47], and then the preoptimized geometries were further precision optimized via density functional theory (DFT) calculations using B3LYP exchange-correlation functional (with empirical dispersion correction of GD3BJ) in conjunction with a 6–31 g (d,p) basis set, which has been wildly used in the study of DESs [48,49]. Moreover, the SMD implicit solvation model was applied in this study for representing the solvation environment of DES catalysts according to Truhlar et al. [50]. The Counterpoise correction was simultaneously adapted to solve the problem of the basis set superposition error (BSSE) [51]. The ESP, vdW potential, noncovalent interaction (NCI) map, atoms-in-molecules (AIM), and electron density difference analyses on the basis of the geometry and wavefunction at the B3LYP (gd3bj)/6–31 g (d,p) level were performed via the Multiwfn 3.8 code developed by Tian Lu [52]. The isosurface maps, 2D scatter and independent gradient model based on the Hirshfeld partition (IGMH) [40] were rendered by Visual Molecular Dynamics (VMD) software version 1.9.3 [53] based on the abovementioned files obtained by Multiwfn.

### 3.6. Statistical Analysis

Each experiment was carried out in triplicate unless otherwise stated; the results were collected and summarized in the form of mean ± standard deviation (SD) values. The analysis of variance (ANOVA) with the least significant difference (LSD) test at *p* ≤ 0.05 was performed via SPSS software (version 25.0, IBM SPSS Statistics, USA).

## 4. Conclusions

In this study, an efficient, high-selectivity, low-cost and sustainable strategy for the synthesis of phytosterol esters was successfully developed with the DESs as the catalyst. Several widely used DESs were screened, and it was found that ChCl/PTSA (1:3) performed best among them. After further optimization, a yield of higher sterol esters (94.1%) and lower level of dehydrated sterols (4.1%) can be obtained in the presence of trace DES catalyst. In addition, the esterification experiments of pine sterols and various fatty acids were carried out, and high product yields were obtained; there was no obvious difference in the yield of sterol esters corresponding to each fatty acid. Compared to the methods reported in the literature, the synthesis method of phytosterol esters in this report had a relatively low reaction temperature, a small amount of catalyst and a light color of the resulting product. Finally, DFT calculations suggested that the H-bonds between ChCl and PTSA decreased the nucleophilic capacity and increased the steric hindrance of the latter, which further prevented the attack of ^β^H and reduced the generation of by-products.

## Figures and Tables

**Table 1:** Catalytic effect of different DESs ^a^.

Entries	DESs	Conversion (%)	Esterification Yield (%)	Dehydrated Sterols Yield (%)	Others (%)
1	ChCl/ZnCl_2_(1:2)	39.5 ± 1.5	36.4 ± 1.9	2.3 ± 0.5	0.8 ± 0.6
2	ChCl/SnCl_2_(1:2)	55.3 ± 1.9	51.3 ± 2.1	3.4 ± 0.3	0.6 ± 0.5
3	ChCl/Benzoic acid (BEN) (1:1)	49.9 ± 0.9	46.7 ± 1.1	2.3 ± 0.6	0.9 ± 0.7
4	ChCl/Oxalic acid (OA) (1:1)	44.2 ± 2.1	40.5 ± 1.6	2.5 ± 1.1	1.2 ± 1.1
5	ChCl/Citric acid (CA) (1:1)	38.5 ± 1.7	35.2 ± 1.5	2.2 ± 0.9	1.1 ± 0.8
6	ChCl/Urea (1:2)	35.9 ± 1.3	33.1 ± 1.3	2.4 ± 0.9	0.4 ± 0.2
7	ChCl/Lactic acid (HL) (1:2)	43.8 ± 1.3	40.8 ± 1.5	2.4 ± 0.8	0.6 ± 0.3
8	ChCl/Sulfosalicylic acid (SSA) (1:2)	95.9 ± 1.8	89.7 ± 1.6	5.3 ± 1.1	0.9 ± 0.7
9	ChCl/PTSA (1:2)	99.9 ± 1.2	65.3 ± 0.6	31.1 ± 0.5	3.5 ± 0.5
10	ChCl/SSA (1:2) ^b^	75.9 ± 1.3	72.0 ± 1.2	3.6 ± 0.6	0.3 ± 0.3
11	ChCl/PTSA (1:2) ^b^	95.0 ± 0.8	90.0 ± 1.1	4.6 ± 0.5	0.4 ± 0.3
12	TBAC/PTSA (1:2) ^b^	92.6 ± 1.5	87.5 ± 1.9	4.4 ± 0.8	0.7 ± 0.2
13	TBAB/PTSA (1:2) ^b^	92.4 ± 1.3	86.8 ± 1.3	4.7 ± 0.7	0.9 ± 0.5
14	ChCl/PTSA (1:3) ^b^	97.9 ± 1.6	92.9 ± 2.0	4.1 ± 0.5	0.9 ± 0.4
15	ChCl/PTSA (1:4) ^b^	98.1 ± 1.5	92.0 ± 1.7	5.3 ± 0.6	0.8 ± 0.6
16	PTSA ^b^	98.7 ± 1.3	88.6 ± 1.7	8.9 ± 0.5	1.2 ± 0.8
17	PTSA ^c^	98.3 ± 1.7	88.7 ± 1.5	8.5 ± 0.5	1.1 ± 0.5

Reaction conditions: ^a^ 2 mmol pine sterols, 4 mmol oleic acid, 7% catalyst (based on the mass of pine sterols), 120 °C, 5 h. ^b^ 2 mmol pine sterols, 4 mmol oleic acid, 1% catalyst (based on the mass of pine sterols), 120 °C, 5 h. ^c^ 2 mmol pine sterols, 4 mmol oleic acid, the amount added is the same as the amount of PTSA in 1% ChCl/PTSA (1:3), 120 °C, 5 h. The relevant raw GC data are shown in Table S1. The detailed composition and sources of the DESs used in this study are shown in Table 4.

**Table 2:** Effect of different fatty acids on esterification ^a^.

Fatty Acid	Conversion (%)	Esterification Yield (%)	Dehydrated Sterols Yield (%)
Lauric acid (C12:0) ^b^	99.4 ± 1.2	94.9 ± 1.0	4.5 ± 0.8
Myristic acid (C14:0)	99.2 ± 1.6	94.5 ± 1.2	4.7 ± 0.7
Palmitic acid (C16:0)	98.7 ± 1.1	94.3 ± 1.3	4.4 ± 1.0
Stearic acid (C18:0)	97.9 ± 0.9	93.6 ± 1.0	4.3 ± 0.7
Oleic acid (C18:1)	98.5 ± 1.0	94.1 ± 1.1	4.1 ± 0.6
Linoleic acid (C18:2)	98.0 ± 1.5	93.6 ± 1.2	4.4 ± 0.5
Linolenic acid (C18:3)	97.9 ± 1.7	93.4 ± 1.5	4.5 ± 0.9

^a^ Reaction conditions: 2 mmol pine sterols, 3 mmol fatty acid, 1.5% catalyst (based on the mass of pine sterols), 120 °C, 3 h. ^b^ The first number in parentheses represents the number of carbon atoms of the fatty acid, and the second number represents the number of carbon–carbon double bonds of the fatty acid.

**Table 3:** The reaction conditions and results of different reported chemical syntheses of phytosterol esters.

Catalyst	Conditions				Result		Reference
	Catalyst Dose (%, Based on Mass of Phytosterols)	Molar Ratio of Fatty Acids to Phytosterols	Reaction Temperature (°C)	Reaction Time (h)	Conversion (%)	Esterification Yield (%)	
H_2_SO_4_	1	1.5:1	170	2	98.2	67.3	[19]
NaHSO_4_	1	1.5:1	170	2	95.4	75.5	[19]
ZnO	1	1.5:1	170	8	84.2	82.9	[19]
Catalyst free	0	3:1	220	4	99.1	94.9	[34]
Catalyst free (microwave irradiation)	0	5:1	180	0.83	95.4	-	[11]
DBSA	20(mol%)	3:1	60	20	95.0	-	[36]
DBSA (microwave irradiation)	20(mol%)	2.5:1	60	1	94.3	-	[37]
[BSO_3_HMIm]Ts	7	2:1	140	2.5	98.4	93.9	[13]
ChCl/SnCl_2_(2:1)	7	3:1	150	4	-	92.3	[22]
ChCl/PTSA (1:3)	1.5	1.5:1	120	3	98.5	94.1	This study

- Not reported.

**Table 4:** Composition of DESs.

Organic Salt	Hydrogen Bond Donor (HBD)	Salt:HBD (Molar Ratio)	Ref
ChCl	ZnCl_2_	1:2	[22]
ChCl	SnCl_2_	1:2	[22]
ChCl	Oxalic acid (OA)	1:1	[41]
ChCl	Benzoic acid (BEN)	1:1	[41]
ChCl	Citric acid (CA)	1:1	[41]
ChCl	Lactic acid (HL)	1:1	[42]
ChCl	Urea	1:2	[43]
ChCl	Sulfosalicylic acid (SSA)	1:2	[44]
ChCl	PTSA	1:2	[44]
TBAC	PTSA	1:2	[44]
TBAB	PTSA	1:2	[29]
ChCl	PTSA	1:3	[28]
ChCl	PTSA	1:4	[45]

## Data Availability

Not applicable.

## Supplementary Materials

The following supporting information can be downloaded at: https://www.mdpi.com/article/10.3390/molecules28030993/s1, Figure S1: MS spectra of the products and dehydrated sterols; Figure S2: The sterol esters synthesis process; Figure S3: Gas chromatogram of reaction mixture when different fatty acids are used as acyl donors; Figure S4: The possible geometries of ChCl/PTSA (1:3) optimized via B3LYP (gd3bj)/6–31g (d,p) method; Table S1: The raw GC data of each entry in Table 1; Table S2: The coordinate of Figure S4f; The detailed experimental procedure of scaled-up experiments of catalyst recovery.
