# Supplementary material for: Selective and Efficient Synthesis of Pine Sterol Esters Catalyzed by Deep Eutectic Solvent
Source: Molecules. 2023 Jan 19;28(3):993. doi: 10.3390/molecules28030993 (PMC9921590; doi:10.3390/molecules28030993)
Supplement: Supplementary file 1 [file molecules-28-00993-s001.zip › molecules-2138283-supplementary.pdf]

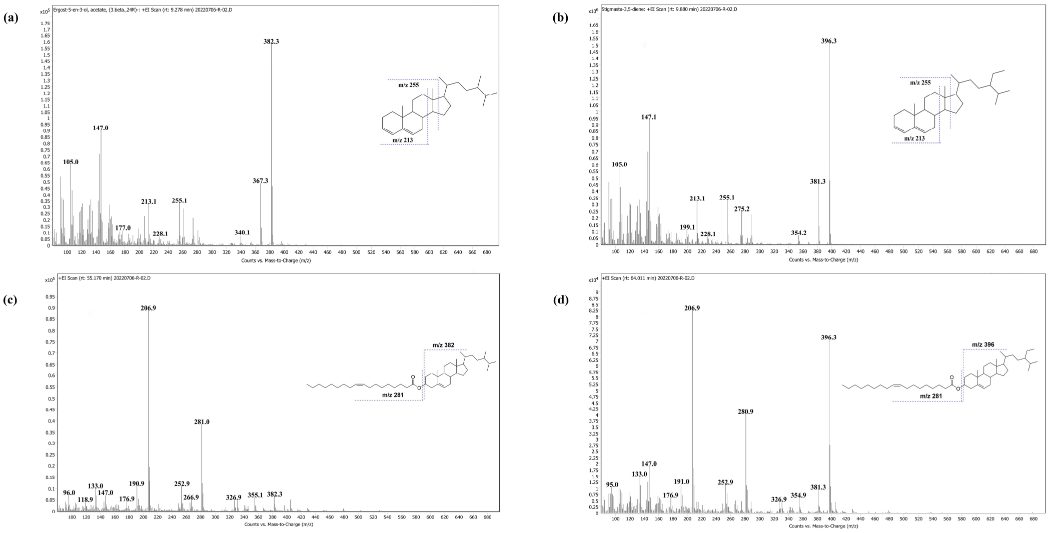

Figure S1. MS spectra of the products and dehydrated sterols. a: the MS spectra of campesterol oleate; b: the MS spectra of β-Sitosterol oleate; c: the MS spectra of dehydration products of campesterol (ergosta-3,5-diene); d: the MS spectra of dehydration products of dehydrated product of β-sitosterol (stigmasta-3,5-diene).

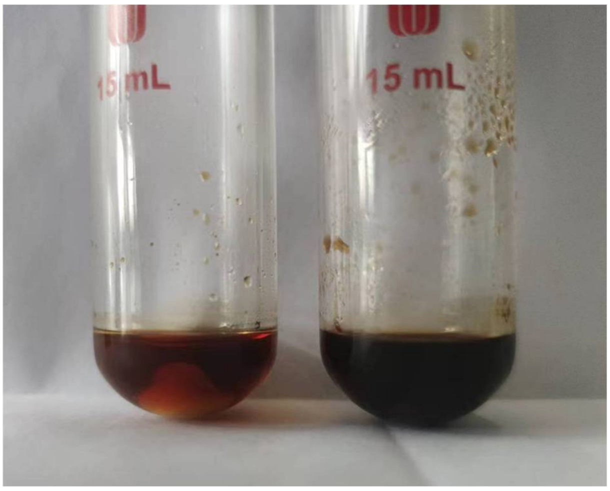

Figure S2. The sterol ester synthesis process. a: Catalyst: ChCl:PTSA (1:3); b: Catalyst: PTSA.

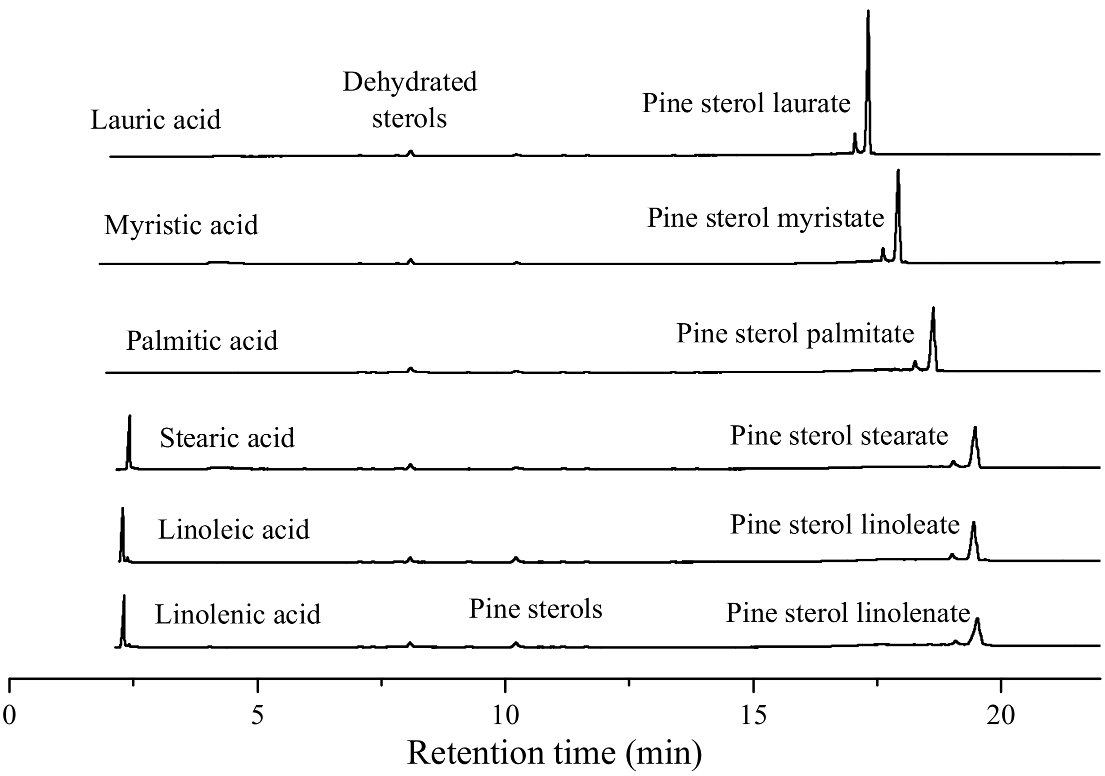

Figure S3. Gas chromatogram of reaction mixture when different fatty acids are used as acyl donors. Reaction conditions: 2 mmol pine sterols, 3 mmol fatty acid, 1.5% catalyst (based on the mass of pine sterols), 120 °C, 3 h.

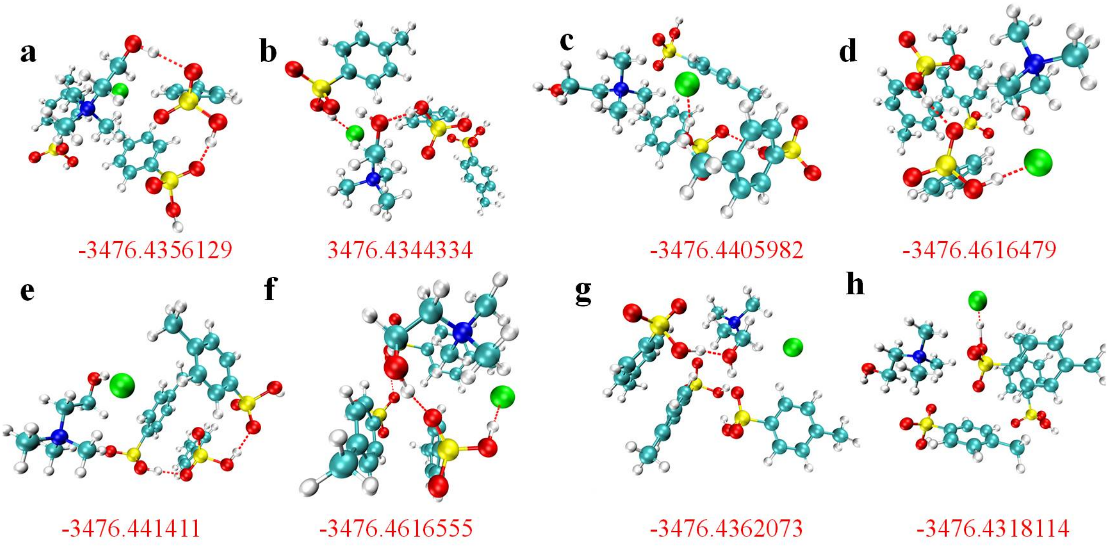

Figure S4. The possible geometries of ChCl/PTSA (1:3) optimized via B3LYP(gd3bj)/6-31g(d,p) method. The single point energy of these geometries was labeled in red color and the unit is Hartree.

Table S1. The raw GC data of each entry in Table 1 [a]

Entries	DESs	Peak area			Sampling amount (mg)
		Dehydrated sterols	Pine sterols	Pine sterol esters	
1	ChCl/$ZnCl_2$(1:2)	93.4	1325	635.4	33.1
2	ChCl/$SnCl_2$(1:2)	127.9	922	883.7	32.0
3	ChCl/BEN (1:1)	58.3	827	612.5	25.0
4	ChCl/OA (1:1)	128	1782	1084	49.0
5	ChCl/CA (1:1)	95.7	1357.7	631	33.6
6	ChCl/Urea (1:2)	88.5	1135	453.6	26.9
7	ChCl/HL (1:2)	76.8	1261.3	761.5	34.7
8	ChCl/SSA (1:2)	148	119	1534	31.3
9	ChCl/PTSA (1:2)	673.7	12.3	1132.1	32.0
10	ChCl/SSA (1:2) [b]	109.4	448.1	1105	28.3
11	ChCl/PTSA (1:2) [b]	144.1	109	1609.4	31.9
12	TBAC/PTSA (1:2) [b]	87.3	186.1	1586	33.2
13	TBAB/PTSA (1:2) [b]	117.9	141.2	1129.3	24.2
14	ChCl/PTSA (1:3) [b]	149	59.6	1808	35.3
15	ChCl/PTSA (1:4) [b]	156.6	55	1625.5	31.7
16	PTSA [b]	164.1	33	1174.5	24.3
17	PTSA [c]	140.5	31.9	1059.8	21.9

Reaction conditions: [a] 2 mmol pine sterols, 4 mmol oleic acid, 7% catalyst (based on the mass of pine sterols), 120 °C, 5 h. [b] 2 mmol pine sterols, 4 mmol oleic acid, 1% catalyst (based on the mass of pine sterols), 120 °C, 5 h. [c] 2 mmol pine sterols, 4 mmol oleic acid, the amount added is the same as the amount of PTSA in 1% ChCl/PTSA (1:3), 120 °C, 5 h.

The external standard curve equations: Dehydrated sterols: y=764.91x+49.758; Pine sterols: y=769.11x+9; Pine sterol oleate: y=413.74-45.706. (y represents the peak area and x represents the mass concentration of the substance in the sample).

Table S2. The coordinate of Figure S4f

C	2.9933	0.5574	2.9542
C	1.6939	0.6158	2.428
C	1.1694	-0.4364	1.6864
C	1.957	-1.5668	1.47
C	3.2442	-1.6642	1.9928
C	3.7529	-0.5982	2.7321
C	3.5603	1.7295	3.711
S	1.3296	-2.8986	0.4752
O	0.4567	-3.7941	1.4402
O	0.4524	-2.2751	-0.5461
O	2.4462	-3.7183	0.0113
H	1.096	1.5074	2.591
H	0.1668	-0.3888	1.2834
H	3.8364	-2.5526	1.8039
H	4.7583	-0.662	3.1393
H	2.8288	2.1373	4.4169
H	4.4576	1.4495	4.2708
H	3.8276	2.531	3.0129
H	-0.3549	-3.2768	1.8632
C	-3.4817	1.0662	2.6128
C	-2.1107	1.1647	2.317
C	-1.6702	1.7785	1.152
C	-2.613	2.3123	0.2666
C	-3.9765	2.2479	0.5394
C	-4.3988	1.6232	1.7148
C	-3.9294	0.3471	3.8565
S	-2.0839	2.913	-1.3233
O	-1.0774	4.1245	-0.9823
O	-1.3148	1.8307	-1.9615
O	-3.221	3.5019	-2.0164
H	-1.3927	0.7113	2.9927
H	-0.6135	1.8259	0.9168
H	-4.6857	2.6739	-0.1617
H	-5.4616	1.5639	1.934
H	-3.539	0.8383	4.7558
H	-5.0205	0.3181	3.9336
H	-3.5419	-0.6786	3.8513
H	-0.2049	3.7642	-0.6742
C	2.7714	-1.2634	-2.4944
C	1.7955	-0.3215	-2.8516
C	1.7022	0.9062	-2.208
C	2.6296	1.1981	-1.2043

C	3.6243	0.2935	-0.8316
C	3.6799	-0.9364	-1.4783
C	2.8265	-2.6106	-3.1636
S	2.5305	2.7503	-0.3715
O	2.6915	3.8484	-1.5784
O	1.1578	2.9934	0.0971
O	3.652	2.9019	0.5493
H	1.0835	-0.5706	-3.631
H	0.9134	1.604	-2.4634
H	4.3096	0.5387	-0.0294
H	4.4183	-1.6691	-1.1686
H	3.6981	-2.6845	-3.8262
H	2.9095	-3.3993	-2.4096
H	1.9269	-2.7931	-3.7569
H	3.644	3.9655	-1.7571
O	-0.6576	-2.6053	-3.084
C	-1.6046	-1.5626	-3.1664
C	-2.9884	-1.9162	-2.6112
N	-3.0833	-2.1056	-1.1069
C	-2.3917	-0.9733	-0.3947
C	-4.5257	-2.0777	-0.699
C	-2.5106	-3.4394	-0.6881
H	-0.126	-2.4936	-2.2699
H	-1.2442	-0.6283	-2.7227
H	-1.7776	-1.3575	-4.2305
H	-3.6762	-1.1015	-2.8578
H	-3.3544	-2.8423	-3.0623
H	-2.5865	-1.0757	0.6711
H	-1.3218	-1.0494	-0.5582
H	-2.7789	-0.0418	-0.7979
H	-4.5767	-2.2975	0.3684
H	-5.0732	-2.8292	-1.2707
H	-4.9283	-1.0826	-0.8945
H	-3.1423	-4.2202	-1.1161
H	-1.4996	-3.5217	-1.0682
H	-2.5071	-3.4729	0.4032
Cl	-1.8343	-2.4437	2.6171

The detailed experimental procedure of scaled-up experiments of catalyst recovery

The experiments of catalyst recovery were carried out in 250 mL three-neck flasks equipped with a magnetic stirring bar to achieve better contact. The reaction was carried out under the optimal conditions: the molar ratio of oleic acid to pine sterols 1.5:1, 1.5% DES dose (based on the mass of pine sterols), reaction at 120°C for 2.5 h. In each reaction cycle, the amount of substrate added was ten times that of the original screening experiment: pine sterols (8.275 g, 0.02 mol), and oleic acid (8.483 g, 0.03 mol). After the reaction, the reaction mixture was transferred to a partition funnel and into an appropriate amount of hot water for extraction. The lower water layer was then concentrated in a rotary evaporator to obtain the DES catalyst, which was dried fully in a vacuum drying oven and used for the next cycle of the reaction.
